# Clinical analysis and medium-term follow-up of simultaneous interventional therapy for compound congenital heart disease in children: a single-center retrospective study

**DOI:** 10.3389/fped.2023.1193136

**Published:** 2023-06-22

**Authors:** Tingting Ran, Lingxin Feng, Mi Li, Qijian Yi, Xu Zhu, Xiaojuan Ji

**Affiliations:** ^1^ Department of Ultrasound, Children’s Hospital of Chongqing Medical University, Ministry of Education Key Laboratory of Child Development and Disorders, National Clinical Research Center for Child Health and Disorders, Chongqing Key Laboratory of Pediatrics, Chongqing, China; ^2^ Department of Cardiovascular Medicine, Children’s Hospital of Chongqing Medical University, Chongqing, China; ^3^ Department of Ultrasound, Chongqing General Hospital, Chongqing, China

**Keywords:** compound congenital heart disease, pediatric, interventional therapy, transthoracic echocardiography, transcatheter

## Abstract

**Objective:**

This study aimed to explore the safety and efficacy of simultaneous interventional therapy for compound congenital heart disease (CCHD) in children.

**Methods:**

In total, 155 children with CCHD who received simultaneous interventional therapy at the Children's Hospital of Chongqing Medical University between January 2007 and December 2021 were included in study. Data on clinical manifestations, transthoracic echocardiography, electrocardiogram, and follow-up were retrospectively analyzed.

**Results:**

The most common type of CCHD was atrial septal defect (ASD) combined with ventricular septal defect (VSD), accounting for 32.3% of the patients. Simultaneous interventional therapy was successfully administered to 151 children (97.4%). The pulmonary gradient of patients with pulmonary stenosis decreased from 47.3 ± 21.9 mmHg to 15.2 ± 12.2 mmHg (*P *< 0.05) immediately after the procedure. One patient had failed PBPV as he had residual PS >40 mmHg post procedure. The right ventricular dimension and left ventricular end-diastolic dimension significantly decreased in the first month after the procedure in patients with ASD combined with VSD. Twenty-five (16.1%) patients had mild residual shunt, which spontaneously disappeared in more than half of these patients 6 months after the procedure. The major adverse events were minimal (*n* = 4, 2.58%), including one patient requiring drug treatment for complete atrioventricular block and three patients receiving surgical treatment because of cardiac erosion, anterior tricuspid valve chordae rupture, and hemolysis, respectively.

**Conclusions:**

ASD combined with VSD is the most common type of CCHD in children, and simultaneous interventional therapy for CCHD in children is safe and effective with satisfactory results. Ventricular remodeling can be reversed in patients with ASD combined with VSD 1 month after the procedure. Most adverse events associated with interventional therapy are mild and manageable.

## 1. Introduction

Compound congenital heart disease (CCHD) is a disease characterized by the concurrent occurrence of two or more prevalent congenital heart diseases (CHD) that can be treated by interventional therapy (1). CCHD can cause more significant changes in cardiac structures and functions due to the prevalence of higher ventricular volume or pulmonary blood flow observed in this disease compared with CHD in the same condition. These changes result in exercise intolerance. Some patients with CCHD have a risk of severe pulmonary hypertension and heart failure with significant mortality. Hence, patients with CCHD should be treated with intervention or surgery. Compared to surgery, with the low mortality rate associated with surgical repair, interventional therapy has become the main treatment method for CHD owing to the availability of percutaneous therapy with less trauma and shorter hospitalization time (2, 3). Several studies have demonstrated the safety and efficacy of interventional therapy in the treatment of CHD (4, 5). However, studies assessing the effects of simultaneous interventional therapy on CCHD are insufficient, leading to difficulties in clinical management. Therefore, we retrospectively analyzed the clinical data of 155 children with CCHD at our center to evaluate the medium-term safety and efficacy of simultaneous interventional therapy.

## 2. Materials and methods

### 2.1. Patients and methods

In total, 155 children who received simultaneous interventional therapy at the Children's Hospital of Chongqing Medical University between January 2007 and December 2021 were included in this study. General characteristics of the study participants are shown in Table 1. Briefly the minimum age and weight were 3 months and 5.5 kg, respectively, whereas the maximum age and weight were 166 months and 40 kg, respectively. The heart function of all patients was classified as class I according to the New York Heart Association (NYHA) functional classification, and they underwent chest x-ray, electrocardiogram (ECG), and transthoracic echocardiography (TTE) before the procedure.

**Table 1:** Patients’ general characteristics (*n* = 155).

Variable	Result
Sex (female), *n* (%)	87 (56.1%)
Age (months)	46.9 ± 29.8 (3–166)
Weight (kg)	14.7 ± 5.5 (5.5–40)
Length of stay (days)	7.2 ± 3.2 (3–34)
Type of CCHD	
ASD + VSD	50 (32.3%)
ASD + PS	35 (22.6%)
ASD + PDA	32 (20.6%)
VSD + PDA	28 (18.1%)
PDA + PS	8 (5.2%)
VSD + PS	1 (0.6%)
ASD + PDA + PS	1 (0.6%)
ASD type	
Secundum central ASD	118 (100.0%)
VSD type	
Perimembranous VSD (PMVSD)	77 (97.4%)
Intracristal VSD	1 (1.3%)
Muscular VSD	1 (1.3%)
PDA type	
Funnel PDA	63 (91.3%)
Tubular PDA	6 (8.7%)
PS type	
Valve stenosis	45 (100.0%)

The inclusion criteria were as follows: (1) age <18 years, (2) a diagnosis of CCHD by TTE and cardiac catheterization and recommendation for device closure or balloon valvuloplasty according to the consensus and other references from previous studies (1–3, 6), and (3) the absence of other cardiovascular malformations requiring surgery. The exclusion criterion was as follows: the presence of infective endocarditis, concomitant hemorrhagic disorders, or thrombocytopenia.

Patients with a body weight >20 kg received intravenous and sacral block anesthesia, whereas the others received intravenous anesthesia. Patients who underwent atrial septal defect (ASD) or ventricular septal defect (VSD) occlusion were administered intravenous heparin (100 IU/kg) during the procedure; their right femoral artery and vein were punctured, and they received interventional treatment after routine left and right cardiac catheterizations. All the operations were performed following established procedures (1, 6).

### 2.2. Materials

The VSD and ASD occluders were provided by Shanghai iShape Memory Alloy (SHSMA) Co. Ltd, Shenzhen Xianjian technological corporation of China, and Beijing Huayishengjie technological corporation of China, devices from SHSMA were most commonly used, followed by Xianjian, in combination, they accounted for about 85%. Patent ductus arteriosus (PDA) occluders were provided by SHSMA Co. Ltd, Shenzhen Xianjian technological corporation of China, Beijing Huayishengjie technological corporation of China and spring coil device of COOK corporation, devices from SHSMA and Xianjian accounted for 72%, and spring coil accounted for 11%. Further, the CRISTAL balloon were provided by Asia Pacific technology and Bart corporation of France.

### 2.3. Follow-up

Patients who received ASD or VSD occluders routinely received oral aspirin (3–5 mg/kg) for 6 months. Follow-up evaluations were performed in all patients. Clinical manifestations and cardiac angiography, TTE, and ECG findings were evaluated to assess the therapeutic effects of the procedure. The severity of adverse events was divided into five levels, as shown in Table 2: grade 1 or 2, without changes or with transient changes in condition that were not life-threatening but may require monitoring or minor interventions; and grade 3, 4, or 5, changes in condition that were life-threatening and required intensive care and further interventions (7, 8). Severe valve regurgitation and pericardial effusion were observed using TTE. Arrythmia was diagnosed by ECG and thrombocytopenia by FBC post interventions.

**Table 2:** Definitions for adverse event severity.

	Severity level	Definition
Low	1. None	No harm, no change in condition, may have required monitoring to assess for potential change in condition with no intervention indicated
	2. Minor	Transient change in condition, not life threatening, condition returns to baseline, required monitoring, required minor intervention
High	3. Moderate	Transient change in condition may be life threatening if not treated, condition returns to baseline, required monitoring, required intervention such as reversal agent, additional medication, transfer to the intensive care unit for monitoring of a serious condition, or moderate trans-catheter intervention to correct condition.
	4. Major	Change in condition, life threatening if not treated, change in condition may be permanent, may have required intensive care unit admission or emergent readmission to hospital, may have required invasive monitoring, required interventions, such as electrical cardioversion or unanticipated intubation or required major invasive procedures or trans-catheter interventions to correct condition.
	5. Catastrophic	Any death, and emergent surgery or heart lung bypass support (ECMO) to prevent death with failure to wean from bypass support.

### 2.4. Statistical analyses

All results are expressed as numbers and percentages for categorical variables and means ± standard deviations with ranges for continuous variables. The Statistical Package for the Social Sciences version 22.0 and GraphPad Prism version 6 were used for data processing. The data of pulmonary gradient (PG) before and after percutaneous balloon pulmonary valvuloplasty (PBPV) were analyzed by paired *t*-test, and a *P*-value of <0.05 was considered statistically significant.

## 3. Results

**Figure 1:**
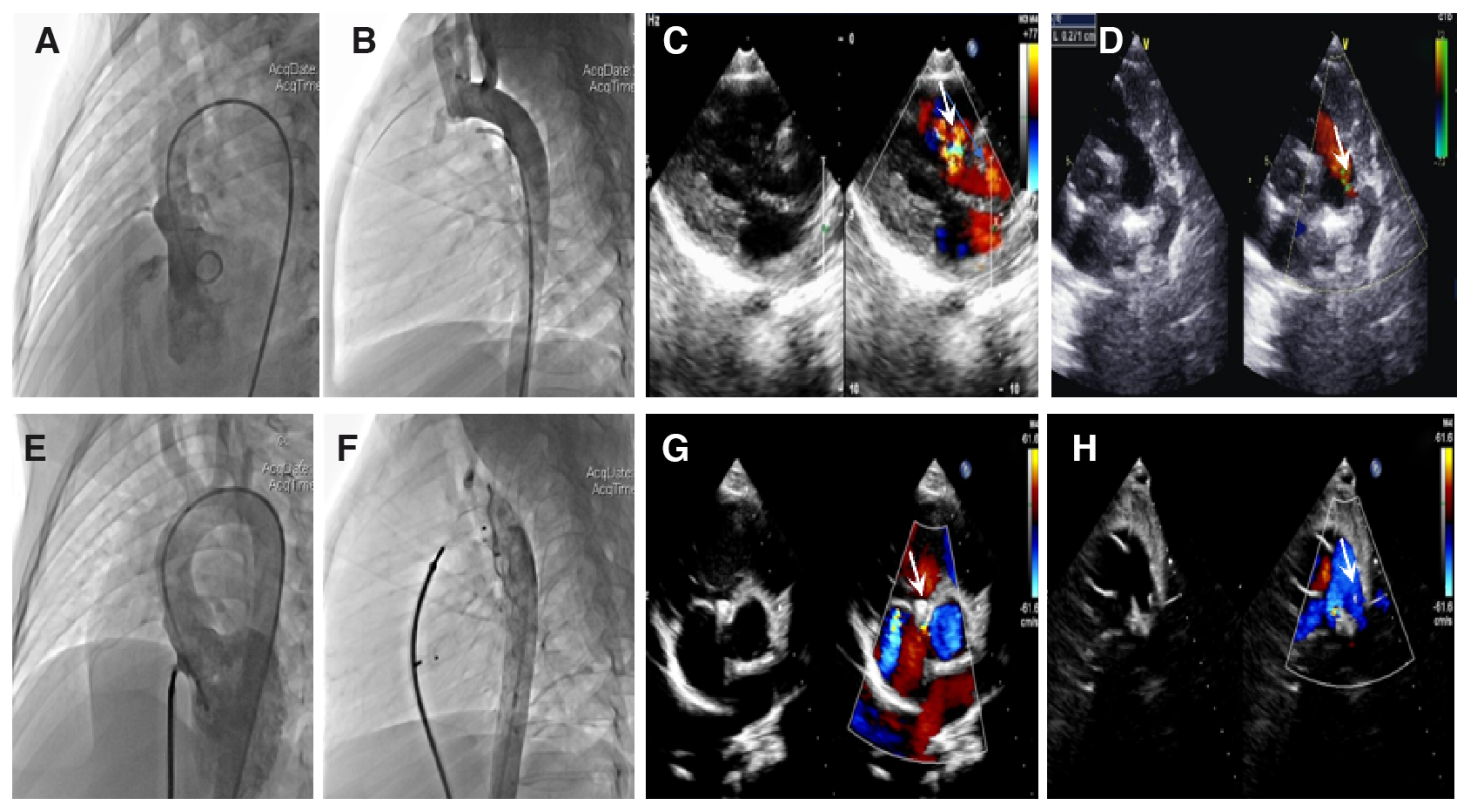
Angiocardiography and echocardiography images in a patient with ventricular septal defect (VSD) combined with patent ductus arteriosus (PDA). Preoperation: (**A**) Long-axis view of the left ventriculography showing a significant left-to-right shunt through the VSD. (**B**) Lateral view of the aortic angiography showing an evident shunt from the aorta to the pulmonary artery through the PDA. (**C**) Parasternal left ventricular long-axis view showing a left-to-right shunt passing through the VSD. (**D**) Parasternal short-axis view at the base of the main pulmonary artery (MPA) and its branches showing a shunt from the aorta to the pulmonary artery through the PDA. Postoperation: (**E**) No evident shunt is detected after VSD occlusion by left ventriculography. (**F**) No aortopulmonary shunt is detected. (**G,H**) Parasternal short-axis view showing that the VSD and PDA occluders are properly placed; no evident shunt is detected. The white arrow points to the defect and occluder.

**Figure 2:**
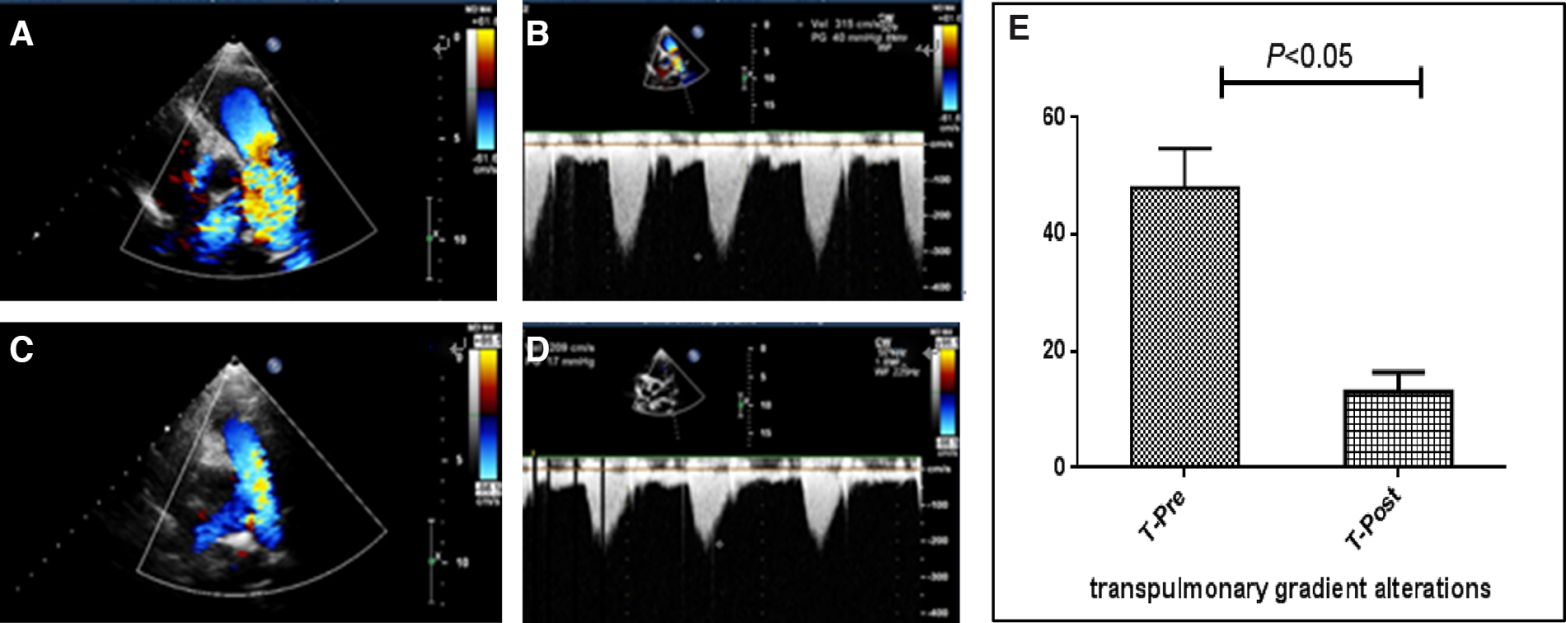
Images of echocardiography and pulmonary gradient (PG) changes in a patient with pulmonary stenosis (PS). Preoperation: (**A**) Parasternal short-axis view at the base with the main pulmonary artery and its branches displaying high-speed flow through the pulmonary valve with color flow Doppler. (**B**) Continuous-wave spectral Doppler showing full envelope spectral Doppler tracing; the measured velocity is approximately 3.1 m/s, or approximately 40 mmHg, as calculated by the simplified Bernoulli equation. Postoperation: (**C**) Color flow Doppler displaying smooth blood flow through the pulmonary valve. (**D**) Continuous-wave Doppler measuring the velocity to be approximately 2.0 m/s, or PG is calculated to be approximately 17 mmHg. (**E**) PG before and after percutaneous balloon pulmonary valvuloplasty is analyzed using a paired t-test, which revealing that PG decreased significantly.

During the procedure, all patients underwent successful simultaneous interventional therapy. The positions of the occluders were well fixed, and no severe residual shunt or valve regurgitation was detected on TTE (Figure 1). TTE revealed a significant change in PG, which imminently decreased from 47.3 ± 21.9 mmHg to 15.2 ± 12.2 mmHg after PBPV (Figure 2). No complete atrioventricular block (cAVB) was found by ECG, and the average time of intervention was 66.7 ± 28.9 min.

Of the 155 patients with available TTE and ECG studies post procedure, the mean follow-up duration was 19.5 months with maximum follow-up time of 84 months. In total, 153/155 (98.7%) of the patients had NYHA class I, and 26/155 (16.8%) had adverse events (Table 3). Among these patients, the incidence rate of grade 1 or 2 adverse events was 84.6%, which was more common in incomplete right bundle-branch block (IRBBB). Two children had iliac artery thrombosis and a femoral artery pseudoaneurysm on vascular ultrasonography and recovered after treatment. Severe adverse events such as deaths, infective endocarditis, cerebrovascular accidents, or device displacement were not observed.

**Table 3:** Adverse events in children after the intervention.

	Number of adverse events according to severity level					Number (*n*, %)
	5	4	3	2	1	
Tricuspid valve chordae tendineae rupture		1				1 (0.65%)
Cardiac erosion		1				1 (0.65%)
Arrhythmia						
Incomplete right bundle-branch block					17	17 (10.9%)
Left anterior fascicular block					3	3 (1.94%)
Complete atrioventricular block			1			1 (0.65%)
Vascular-related						
Iliac artery thrombosis				1		1 (0.65%)
Pseudoaneurysm				1		1 (0.65%)
Hemolysis		1				1 (0.65%)

Of the 45 patients who underwent PBPV, PG decreased to normal in 97.8% and didn’t increase during follow-up. In one 6-year-old patient was diagnosed with PS combined with PDA and ASD, the diameter of the narrowest part of pulmonary valve was 5.8 mm, the valve annulus was 15.8 mm, the PG was 82 mmHg before the procedure, the ratio of balloon/valve (BAR) was 1.5 during the procedure, and the PG decreased to 60 mmHg periprocedural and increased to 68 mmHg and 96 mmHg at 1 and 3 months, respectively.

Right ventricular dimension (RVD) and left ventricular end-diastolic dimension (LVEDd) were measured at the end of diastole using M-mode at the level of the mitral valve leaflet tips in the parasternal left ventricular long-axis view. Patients with ASD combined with VSD, ASD combined with PS, and PDA combined with PS were diagnosed with right ventricular dilation using TTE before the procedure, and the mean RVD decreased significantly at 1, 3, and 6 months after the procedure. Furthermore, a significant decrease in mean LVEDd was observed in patients with ASD combined with VSD and VSD combined with PDA simultaneously. The other types of CCHD showed no significant changes in the RVD and LVEDd during the follow-up period (Table 4).

**Table 4:** Changes in RVD and LVEDd.

Type of CCHD	RVD (mm)				
	ASD + VSD	ASD + PS	VSD + PDA	ASD + PDA	PDA + PS
Preprocedure	12.6 ± 3.1	16.7 ± 3.7	12.2 ± 2.2	14.2 ± 4.1	13.3 ± 1.6
1 month postprocedure	11.8 ± 2.8^a^	13.9 ± 2.2^a^	12.3 ± 1.4	12.0 ± 2.0	11.3 ± 1.4^a^
3 months postprocedure	11.4 ± 1.2^a,^^b^	12.7 ± 2.6^a^	11.4 ± 0.5^b^	13.5 ± 2.4^b^	11.0 ± 0.8^a,^^b^
6 months postprocedure	11.3 ± 0.6^a,^^b,^^c^	12.7 ± 1.8^a,^^c^	11.5 ± 0.6^b,^^c^	13.2 ± 3.4^b,^^c^	15.0 ± 1.4^b,^^c^
Type of CCHD	LVEDd (mm)				
	ASD + VSD	ASD + PS	VSD + PDA	ASD + PDA	PDA + PS
Preprocedure	35.2 ± 4.1	30.2 ± 3.5	39.8 ± 3.6	31.5 ± 5.2	28.7 ± 6.2
1 month postprocedure	32.3 ± 3.7^a^	31.0 ± 3.8	34.5 ± 4.1^a^	30.2 ± 4.1	28.2 ± 5.0
3 months postprocedure	32.3 ± 2.2^a,^^b^	31.0 ± 3.1^b^	33.3 ± 2.8^a,^^b^	30.3 ± 3.9^b^	28.5 ± 3.4^b^
6 months postprocedure	32.7 ± 2.6^a,^^b,^^c^	31.2 ± 2.7^b,^^c^	32.7 ± 3.4^a,^^b,^^c^	30.8 ± 3.2^b,^^c^	28.5 ± 3.5^b,^^c^

^a^ *P *< 0.05, compared with preprocedure.

^b^ *P *> 0.05, compared with 1 month postprocedure.

^c^ *P *> 0.05, compared with 3 month postprocedure.

The frequency of mild residual shunt 1 day after the procedure was 16.1% (25 of 155), as measured using TTE, and no changes in urine color were found. Among them, thirteen patients were diagnosed with ASD, including seven patients with more than two ASDs, which mostly occluded by SHSAM and Xianjian. Ten patients were diagnosed with PMVSD, which were occluded by SHSAM, and three with PDA. Six months after the intervention, a mild residual shunt was observed in nine patients, who were diagnosed with PMVSD (three patients) or ASD (six patients, including four patients with more than two ASDs). None of the patients developed moderate or severe residual shunt during the follow-up period.

In total, 129 (84.5%) patients had no arrhythmias after the intervention. ECG revealed left anterior fascicular block in three patients and IRBBB in 17 patients who had no symptoms during the follow-up period. One patient developed cAVB 2 days after the procedure and was converted by using hydrocortisone. His record was reviewed, and it was found that he was diagnosed with perimembranous ventricular septal defect with aneurysm and three defects of 3.6, 2.9, and 2.5 mm in size. A 10-mm-diameter device produced by Xinjian Corporation was selected for occlusion.

Three patients underwent surgery because of severe adverse events. One 12-year-old patient with ASD and PDA occlusion had palpitation, oliguria, and decreased blood pressure for half a day after the procedure, had severe tricuspid regurgitation (TR) and moderate to massive pericardial effusion was observed by TTE, resultingly, the patient underwent surgery, and had erosion of the left atrium. The size of the ASD was 4 mm, the diameter of the device was 10 mm, and the ASD occluder was produced by the Shanghai Shape Memory Alloy Materials Corporation. Another patient experienced severe TR after ASD and PDA occlusion, which persisted for 2 years with cardiac insufficiency, had a ruptured anterior tricuspid valve chordae tendineae, and received tricuspid valvuloplasty. The third patient with VSD combined with PDA presented with hematuria, ecchymosis, thrombocytopenia (minimum 7 × 10^9^/L), and severe TR 1 day after the procedure. There was no improvement after symptomatic treatment, and the patient underwent surgery to remove the occluders and underwent VSD patch and PDA ligation. The PDA was 3.8 mm, and the size of the device was 10 mm.

## 4. Discussion

To the best of our knowledge, CHD is one of the most common congenital malformations in children. Approximately 50% of children with VSD have other heart malformations, including 17% of children with ASD and 6% of children with PDA. Children with ASD also have PS (10%), VSD (5%), and PDA (3%) (9). In our study, ASD combined with VSD was the most common type of CCHD, followed by ASD combined with PS, similar to the findings of Wang et al. (10). The combination of the three types of CHD was rare, accounting for only 0.6% of the patients in this group. Compared to CHD, CCHD always manifests more complicated cardiac hemodynamics, causing impaired functional capacity earlier in children. Percutaneous interventional therapy has become the preferred strategy as its device-related mortality rates are low and adverse events are well described. Thus, this study reports on a larger series of children receiving simultaneous interventional therapy for CCHD to evaluate its efficacy and safety.

Currently, percutaneous interventional therapy for CCHD is not a simple addition of techniques. Selecting a reasonable order for this procedure is crucial. We followed two principles: the difficult first followed by the easy and the complex first followed by the simple. Generally, PBPV is followed by VSD, ASD, and PDA occlusions. These principles are also applicable to the combination of three types of CHD. Simultaneous interventional therapy is safe and effective in children, with a procedural success rate of 97.4%.

PS can cause right atrial and ventricular volume overload, resulting in right ventricular hypertrophy and impairment of diastolic and systolic functions, even causing irreversible right heart failure. After PBPV, both hemodynamic and cardiopulmonary functions tend improve. A previous study found that patients treated with PBPV had an exercise capacity similar to that of healthy controls and that the long-term prognosis of patients was excellent, with rare adverse events (11). In this study, one patient had failed PBPV as he had residual PS >40 mmHg post procedure. We reviewed his medical record and found that he had high initial PG. Which was indipendently associated with residual PS and repeat intervention in a multicenter study following PBPV over 25 years (12).

Owing to the elimination of cardiac overload, the dilated right ventricle significantly decreases in patients with ASD combined with VSD and ASD combined with PS as assesed 1 month after the procedure, and the dilated left ventricle decreases in patients with VSD combined with ASD and VSD combined with PDA simultaneously, demonstrating that the ventricle can reverse remodeling and the procedure prevents deterioration in exercise capacity (13). In addition, remodel may be age dependent and affect heart function after ASD closure, and children may require a shorter time to remodel than older patients (14).

In our series, 12.7% of the patients had a mild residual shunt after VSD closure, the type of VSD were mostly perimemberous and occluded by devices from SHSAM. Additionally, 11.5% of the patients had a mild residual shunt after ASD closure; among them, 61.5% of patients had two or three ASDs, which were mostly occluded by devices from SHSAM and Xianjian. They decreased by half at 6-month follow-up, indicating that the mild residual shunt can disappear spontaneously, which is consistent with the findings of Santhanam et al. (15).

Although the success rate of periventricular device closure was stable in previous reports (16, 17), more attention should be paid to postprocedural arrhythmias, with cAVB being the most serious subtype that requires immediate treatment. Generally, cAVB appears within 1 week of implantation (18). In this cohort, one (1.26%) patient with PMVSD with aneurysm, having three defects (maximum size, 3.6 mm) that were occluded with a 10-mm-diameter device produced by Xianjian, had early-onset cAVB 2 days after the procedure and returned to normal under medication treatment. The distribution of the cardiac conduction system being adjacent to the PMVSD boundary, and mechanical compression and associated inflammatory edema of the conduction system are its possible mechanisms. Moreover, some literature has shown a few patients had late-onset or reappearing cAVB, with cAVB appearing as late as 9 years after the procedure (16). Thus, close observation of a patient’s ECG during the perioperative period and follow-up is required, possibly in the long-term.

Cardiac erosion is a rare but serious adverse event after ASD closure, the risk of erosion ranges from approximately 0.04% to 0.3%, and the locations are mostly in the aorta and left or right atrium. A recent case–control study of 125 erosions after the transcatheter closure of ASD found that the relative risk factors for erosion included deficiency of the aortic rim, a device >5 mm larger than the ASD diameter, and smaller weight:device size ratio (19, 20). In our study, one (0.85%) patient had cardiac erosion located in the left atrium, but it was not deficiency of the aortic rim. We thought that it was likely due to an oversized device, highlighting the importance of a comprehensive evaluation before the procedure and reexamination by TTE in time, especially when pericardial effusion is found. One case of tricuspid valve chordae tendineae rupture after ASD and PDA closure was considered to be related to delivery wire injury to the chordae tendineae when the pathway was established through the tricuspid valve to occlude the PDA, although there were no similar reviews about it. Some reports have found tricuspid injuries after the transcatheter closure of VSD, especially in pseudoaneurysms with a large area and the right ventricular disc of the occluder in poor formation (21). The procedure should be standardized and gentle during the intervention, and the causes of new severe TR after the procedure should be carefully investigated to avoid damage to cardiac function by surgical treatment. Hemolysis is related to platelet aggregation and destruction caused by large occluders and residual shunt after PDA occlusion and is primarily due to the mechanical injury of red blood cells. This mostly occurs within 24 h after the procedure. If hemolysis progresses, surgery is recommended to remove the occluder. Hence, close monitoring of urine color and routine follow-ups are necessary.

## 5. Conclusion

ASD combined with VSD is the most common type of CCHD in children. On the premise of strictly following the interventional therapy guidelines and the operation sequence, we conclude that simultaneous interventional therapy is effective and safe for children with CCHD. Ventricular remodeling can be reversed 1 month after the procedure in patients with ASD combined with VSD. Most adverse events associated with interventional therapy are mild and manageable.

### 5.1. Limitations

Our study has some limitations. First, the number of patients was relatively small, and this was a single-center study that could not effectively represent all children in the=region. Second, a mid-term follow-up period was not adopted for all patients, and the follow-up period was relatively short. Third, this study did not include a surgery group to compare the effects and adverse events. Finally, this was a retrospective study that relied on available medical records, prospective studies, and discussions that had not been conducted.

## Data Availability

The raw data supporting the conclusions of this article will be made available by the authors, without undue reservation.
